# Type I Kounis Syndrome after Protracted Anaphylaxis and Myocardial Bridge—Brief Literature Review and Case Report

**DOI:** 10.3390/diagnostics10020059

**Published:** 2020-01-21

**Authors:** Liviu Ghilencea, Mihaela Roxana Popescu, Ileana Maria Ghiordanescu, Cristina Conea, Mihai Melnic, Andreea Catarina Popescu

**Affiliations:** 1 Cardiology Division, Elias Emergency University Hospital, 011416 Bucharest, Romania; 2 Cardiothoracic Pathology Department, Carol Davila University of Medicine and Pharmacy, 020021 Bucharest, Romania; 3 Allergology Compartment, Elias Emergency University Hospital, 011416 Bucharest, Romania

**Keywords:** Kounis syndrome, insect venom, anaphylactic shock, allergic infarction, challenging diagnosis, myocardial bridging, acute coronary syndrome

## Abstract

The term allergic angina, introduced for the first time by Nicholas Kounis in 1991, initially referred to the coexistence of acute coronary syndromes with allergy or hypersensitivity. At present, it is believed that Kounis syndrome is a particular case of systemic disease, with multiorgan arterial involvement generated during immediate hypersensitivity reactions. Myocardial bridging (MB), a condition that can induce coronary artery spasm, has long been regarded as a benign condition. Since both pathologies are associated with arterial spasm, Kounis syndrome and MB are considered to be confounding pathologies for acute coronary syndromes, and their association is quite a rare finding. To date, there are no precise data on the epidemiology, and the population affected by Kounis syndrome seems to be highly heterogeneous. Since this is a rare disease, even less is known about possible different phenotypes, including MB overlap. We report a case of type I variant Kounis syndrome associated with MB with no evidence of coronary artery disease, occurring as late presentation, following a severe systemic reaction (anaphylaxis) induced by a Hymenoptera sting. At present, only two other cases of type I and one case of type II Kounis syndrome occurring in patients with myocardial bridging have been described.

## 1. Introduction

Necrotizing arteritis and periarteritis of the coronary arteries in humans associated with serum disease after exposure to pneumococcal and tetanus antitoxin were first described in 1938, and several similarities with anaphylactic reactions induced in animals by foreign serums have been observed [1]. In was not until 1950 that the first case of an acute coronary syndrome associated with an allergic reaction due to penicillin was reported [2]. In 1991, Nicholas Kounis described the relationship between allergies and acute coronary syndromes [3] and used the terms “allergic angina” and “allergic infarction” to define the syndrome. Today, there are over 300 Kounis syndrome cases described in medical literature [4]. Additionally, three types of Kounis syndrome have been characterized [5]. The type I variant includes patients with no history of coronary artery disease (CAD) experiencing coronary vasospasm with or without progression to myocardial infarction with positive or negative cardiac biomarkers. Type II Kounis syndrome includes patients with preexisting atheromatous disease, in which an allergic insult renders the plaque unstable, leading to its erosion or rupture. The last variant of Kounis syndrome, type III, includes patients in whom stent thrombosis occurs in response to an allergic reaction.

No race, age group, or geographic predisposition for Kounis syndrome has been observed, and information on the true prevalence and incidence of Kounis syndrome and its variants, based on large prospective trials, is generally lacking.

**Figure 1:**
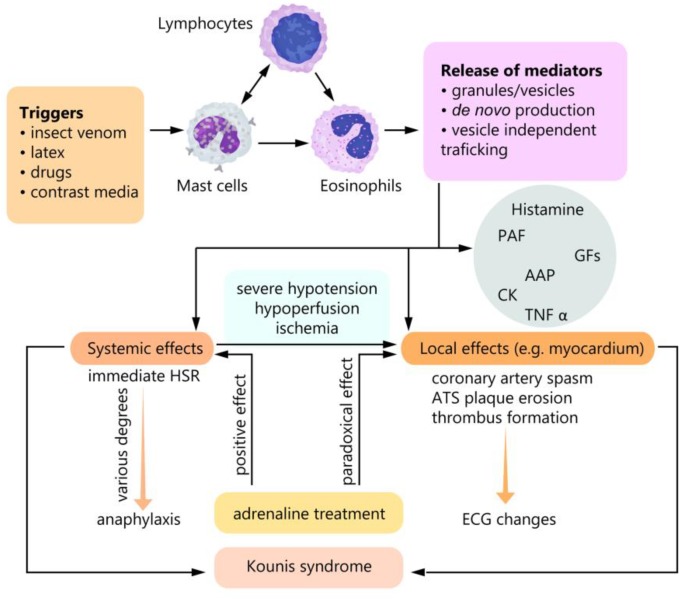
Kounis syndrome pathophysiology. The inflammatory cascade induced by triggers, through activation of mast cells, lymphocytes, and eosinophils, leads to systemic and local effects. The systemic effects have a potential impact on the myocardial tissue through hypotension, resulting in hypoperfusion and subsequent ischemia. Adrenaline can control the systemic response but has a paradoxical effect in the myocardial tissue (favorizes thrombus formation). Both the local and systemic effects can play a role in the generation Kounis syndrome. Arachidonic acid products (AAP), atherosclerotic (ATS), cytokines (CK), growth factors (GFs), hypersensitivity reaction (HSR) [12], platelet-activating factor (PAF).

The main cell type driving the inflammation in Kounis syndrome is represented by the mast cells, which, in certain conditions, release mediators by several molecular pathways: Exocytosis of large dense granules, de novo production of mediators; and pathways independent of vesicle trafficking [6,7]. An immune IgE-dependent or non-immune mechanism can induce degranulation. Histamine, platelet-activating factors (PAFs), cytokines, and the products of the arachidonic acid [8] have been linked to the development of the coronary artery spasm and atheromatous plaque erosion or rupture (see Figure 1). Interestingly, eosinophils seem to bear importance in mast cell activation, as suggested by the peripheral eosinophilia and myocardial eosinophilic infiltration, which are common in Kounis syndrome [4,9,10,11].

The most frequent triggers for Kounis syndrome are drugs (metamizole [13], beta-lactam antibiotics: ampicillin-sulbactam [13,14,15], penicillin [2], cefuroxime [16,17,18], and other antibiotics, such as fluoroquinolones—levofloxacin [19]), contrast agents (gadolinium derivates [20,21]), insect venoms and snake venoms (Hymenoptera, cobra [22,23,24,25]), certain foods (tuna fish [26]), and proteins present in the natural latex [11]. Additionally, predisposing medical conditions, such as myocardial bridge (MB) [27], and atopic conditions have been described. The exact importance of triggers vs. preexistent pathology in driving Kounis syndrome remains to be established.

MB, a condition characterized by an intramyocardial path of the epicardial coronary artery, which can induce coronary artery spasm, is, most of the time, incidentally found during coronary angiography [28]. Some patients may be admitted to emergency units with typical or atypical angina pectoris, ST elevation, or non-ST elevation myocardial infarction (MI). In symptomatic patients with MB, the management is usually medical (including beta-blockers and calcium channel blockers) and rarely surgical (myomectomy) or invasive (stent deployment). The coexistence of MB and Kounis syndrome is very rare. Kounis syndrome can mimic other elusive diseases, such as takotsubo cardiomyopathy [29], also known as stress (catecholaminergic) cardiomyopathy. To complicate things even more, there are case reports with an overlap between these diseases (Kounis syndrome-takotsubo, Kounis-MB).

## 2. Case Presentation

A 63-year-old smoker (25 pack-years) presented to our emergency department with severe chest pain. The patient was not on any current medication and did not have any previously known cardiovascular disease. At 48 h prior to the presentation, several minutes after a wasp sting, an episode of dizziness, lightheadedness, and blurred vision occurred. Treatment with Epinephrine and Hydrocortisone was administrated in another emergency department, where the diagnosis of probable anaphylaxis induced by wasp sting was made. Severe chest pain occurred 48 h later, and the patient was transported to our hospital’s Emergency Room. He was admitted to the Emergency Cardiology Unit, with a preliminary diagnosis of ST-elevation myocardial infarction (STEMI). The pain, described as constrictive, radiating to the shoulder and neck, had a sudden onset, associated nausea, and dyspnea that lasted for several minutes, spontaneously subsided, and then reappeared two days later. The research has obtained the patient’ consent.

**Figure 2:**
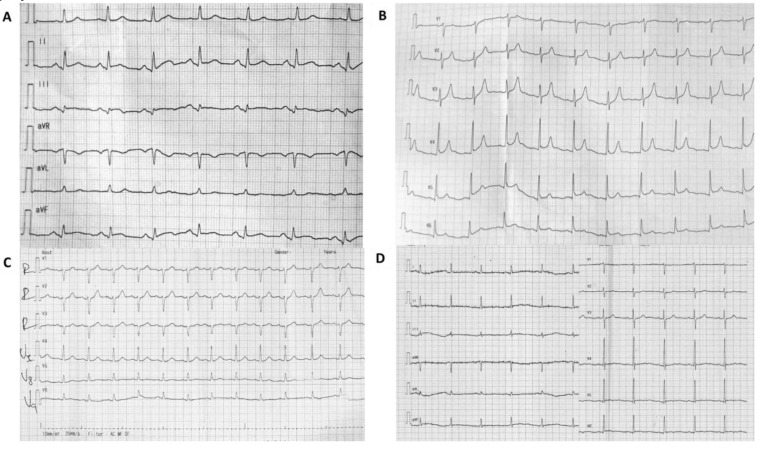
ST-segment evolution. (**A**–**C**) Initial electrocardiogram (ECG) at the emergency department, ST elevation in inferior and lateral leads. (**D**) ECG at discharge, flat ST segment with biphasic T waves.

The electrocardiogram (ECG) performed upon presentation showed normal sinus rhythm of 95 bpm and ST-segment elevation in the inferior and lateral leads (see Figure 2). Furthermore, the transthoracic echocardiography (TTE) confirmed the presence of hypokinesia of the basal septum and the lateral basal wall. Cardiac enzymes were determined and revealed an initial serum troponin I level of 2.02 ng/mL (reference value, <0.02 ng/mL); thus, the patient was referred for urgent angiography.

**Figure 3:**
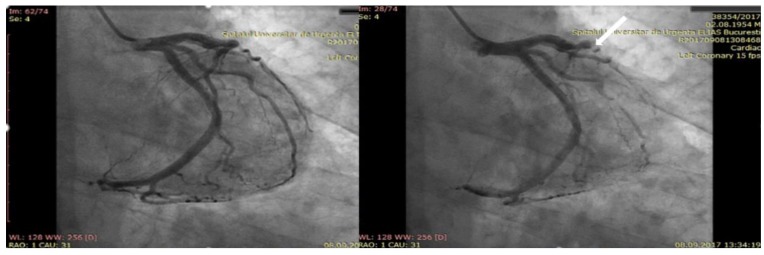
Diagnostic coronary angiogram. The coronary angiogram reveals no apparent coronary lesions. The white arrow indicates myocardial bridging on the medial segment of the left anterior descending.

The diagnostic coronary angiogram depicted normal coronary arteries (see Supplementary Materials) and hemodynamically irrelevant MB at the level of the left anterior descending coronary artery (Figure 3). The MB was located on another artery than the one that appeared to be responsible for myocardial ischemia. The troponin dynamics followed a pattern typical for myocardial infarction, with a rise up to 16 ng/mL shortly after admission and a gradual decline afterward. The total eosinophil count (0.09 x 10^3^/µL) was within the normal range, and inflammatory biomarkers showed a sharp rise (CRP (C reactive protein) = 50.5 mg/L, NR (normal range): 0.2–11 mg/L; fibrinogen = 577 mg/dl, NR: 238–498 mg/dl).

The patient was initially treated as STEMI, with loading doses of dual antiplatelet (aspirin and clopidogrel). Due to the absence of coronary lesions on angiography, upon discharge, he was prescribed only aspirin, together with a statin, a calcium channel blocker, and five days of medication with steroids and antihistamines. He reports not being compliant with the prescribed medication from discharge. On follow-up, he did not experience other episodes of chest pain or systemic reactions. Currently, the patient is being investigated for the presence of specific IgE against wasp venom, and allergen immunotherapy with wasp venom is considered.

## 3. Discussion

All presentations suggesting a Kounis syndrome should be managed as acute coronary syndromes (ACSs). Conversely, all ACSs without coronary lesions and with a clinical context, suggesting a hypersensitivity reaction, should undergo the differential diagnosis of Kounis syndrome. This conduct is necessary until the diagnostic angiogram proves normal coronary arteries, especially since the paraclinical diagnostic of Kounis syndrome can be challenging, as histamine has a short half-life and circulation time of only 8 min (which renders it unpractical to use as a biomarker for acute allergic reactions). Tryptase should be measured 30 min after the onset of symptoms and later every 30 min during the following 2 h [12]. Nevertheless, raised tryptase levels could indicate atheromatous plaque instability [30]; thus, it is advisable to be measured whenever accessible.

In our reported clinical case, a diagnostic of type I variant Kounis syndrome induced by wasp sting and associated with MB was made based on (1) the clinical picture suggesting an IgE-mediated protracted immediate severe reaction to wasp venom, (2) the ECG ST-segment elevation in the inferior and lateral leads, (3) the troponin dynamics typical for myocardial infarction, (4) the lack of previously known cardiac disease, (5) the presence of asymptomatic MB uncovered by angiography, which, interestingly, was located on another artery than the one that appeared to be responsible for the myocardial ischemia, and (6) the clinical evolution on the follow-up with no later recurrence of the symptoms in the absence of the specific trigger.

In the presented case, considering that the patient had been managed initially in another setting, where he received emergency treatment and came to our hospital 48 h after the onset of symptoms, tryptase could not be measured. The search for suitable Kounis syndrome biomarkers, including prostaglandin D2, CD63, carboxypeptidase, and interleukin 4 and 6, CRP is ongoing [31]. Every new clinical case reported could add information to common characteristics, thus facilitating, in time, the generation of a “Kounis marker panel”, which would allow more straightforward diagnostic, better definition of phenotypes, and improved management.

## 4. Conclusions

The diagnostic of Kounis syndrome is challenging and can be complicated by the presence of concomitant cardiac anatomical variants. In our paper, we illustrate, through the means of a case presentation, the peculiarities arising from such an association. To the best of our knowledge, this is the third case reported in the literature of Type I Kounis syndrome overlapping with preexisting MB leading to acute ST-elevation MI. When dealing with such elusive diseases, every newly reported case could add to our understanding of their complex mechanisms.

## Supplementary Materials

The following are available online at https://www.mdpi.com/2075-4418/10/2/59/s1, Video S1: Right coronary artery on coronarography. No visible lesions, Video S2: Circumflex coronary artery on coronarography. No visible lesions.
